# Lactate transporters and vascular factors in HPV-induced squamous cell carcinoma of the uterine cervix

**DOI:** 10.1186/1471-2407-14-751

**Published:** 2014-10-08

**Authors:** Céline Pinheiro, Eduardo A Garcia, Filipa Morais-Santos, Cristovam Scapulatempo-Neto, Allini Mafra, Renske DM Steenbergen, Enrique Boccardo, Luisa L Villa, Fátima Baltazar, Adhemar Longatto-Filho

**Affiliations:** Life and Health Sciences Research Institute (ICVS), School of Health Sciences, University of Minho, Braga, Portugal; ICVS/3B’s - PT Government Associate Laboratory, Braga/Guimarães, Portugal; Barretos School of Health Sciences, Dr. Paulo Prata – FACISB, Barretos, São Paulo, Brazil; Molecular Oncology Research Center, Barretos Cancer Hospital, Barretos, Sao Paulo, Brazil; Department of Pathology, Barretos Cancer Hospital, Barretos, São Paulo, Brazil; Department of Pathology, VU University Medical Center, Amsterdam, The Netherlands; Microbiology Department, Institute of Biomedical Sciences, University of Sao Paulo, Sao Paulo, Brazil; Faculty of Medical Sciences of Santa Casa of Sao Paulo, Sao Paulo, Brazil; Department of Radiology and Oncology, School of Medicine, University of Sao Paulo, ICESP, Sao Paulo, Brazil; Instituto Nacional de Ciência e Tecnologia do HPV (INCT-HPV), Sao Paulo, Brazil; Laboratory of Medical Investigation (LIM-14), School of Medicine, University of Sao Paulo, Av. Dr. Arnaldo, 455 - Cerqueira César 1246-903, Sao Paulo, Brazil

**Keywords:** Angiogenesis, Cervical carcinoma, Hypoxia, Lymphangiogenesis, Metabolic reprogramming, Monocarboxylate transporter, VEGF

## Abstract

**Background:**

Tumour microenvironment is a fundamental aspect of tumour behaviour, modulating important events as cancer cell migration and invasion, as well as angiogenesis and metastisation. Among other microenvironment features, hypoxia and acidity play important roles in this modulation. As the metabolic reprogramming of cancer cells induces extracellular acidity, which in turn induces angiogenesis, and hypoxia induces both the metabolic reprogramming and angiogenesis, the present study aims to evaluate the immunohistochemical expression of a variety of metabolic and vascular markers as common targets of the hypoxic microenvironment in a series of cervical squamous cells carcinoma, as well as using an *in vitro* 3D culture model.

**Methods:**

Immunohistochemical expression of MCT1, MCT4, CD147, GLUT1 and CAIX was assessed in a series of 28 chronic cervicitis, 34 LSIL, 29 HSIL, 38 cases of squamous cells carcinoma (SCC), as well as in *in vitro* 3D culture of keratinocytes expressing HPV genes. Furthermore, VEGF family members’ expression was assessed in the SCC cases. The expression profiles were associated with patients’ clinicopathological parameters.

**Results:**

We found an increase of MCT4 expression along progression to malignancy in cervical samples. Also, MCT4 was associated with CD147 and CAIX expression. VEGF-A expression was more frequently found in cases without MCT1 expression. Both MCT4 and CD147 were more frequently expressed in younger patients at diagnosis while no associations were found between VEGF family and clinicopathological parameters. Finally, we show evidence for the upregulation of MCT4, as well as CD147 and CAIX, after HPV transfection.

**Conclusions:**

The results herein presented point at MCT4 as a promising therapeutic target in squamous cells carcinoma of the uterine cervix. Importantly, we show a possible association between lactate transport and angiogenesis, which should be further explored.

## Background

Monocarboxylate transporters (MCTs) are a family of 14 members and isoforms 1–4 are responsible for the co-transport of monocarboxylates with a proton [1]. They are of extreme importance in the cancer context since they perform a key dual role in the metabolic homeostasis of cancer cells. It is widely known that cancer cells rely on the conversion of glucose into lactate, even in the presence of oxygen, for energy and anabolic intermediates production, a phenomenon known as the Warburg effect [2]. In this context, MCTs, especially MCT1 and MCT4, are crucial for the cellular export of the high amounts of lactate resultant from the high glycolytic activity. Besides, they are also important contributors for intracellular pH maintenance, by partially exporting the protons resultant from the metabolic activity, therefore avoiding intracellular acidification and, consequently, cell death. Importantly, both MCT1 and MCT4 require CD147 as an ancillary protein for correct plasma membrane location and activity [3].

Hypoxia has become a central subject in tumour physiology and cancer treatment. It is primarily a pathophysiologic consequence of oxygen diffusion limitations, due to exacerbated cell proliferation as well as inadequate and chaotic angiogenesis [4]; however, evidence from experimental and clinical studies point to a fundamental role of hypoxia in tumour dissemination, progression and resistance to therapy [5]. In cervical cancer, hypoxia was shown to be associated with shorter 5-year survival for FIGO stage III patients [6]. Importantly, hypoxia, through the transcription factor hypoxia inducible factor 1 alpha (HIF-1α), is the major regulator of cancer cell metabolism, stimulating the Warburg effect [7]. Actually, glucose transporter 1 (GLUT1) and carbonic anhydrase IX (CAIX), which are important players in the hyper-glycolytic and acid-resistant phenotype of cancer cells, are HIF-1α targets [8, 9]. Other important targets of HIF-1α regulation include MCT4 and vascular endothelial growth factor (VEGF) [10].

The VEGF family is formed by a group of highly conserved factors regulating vasculogenesis, haematopoiesis, angiogenesis, lymphangiogenesis and vascular permeability [11], and some of the members are thus considered important therapeutic targets [12, 13]. In mammals, the family comprises VEGF-A, VEGF-B, VEGF-C, VEGF-D and placental growth factor (PlGF). VEGF-A is primarily related to angiogenesis while VEGF-C and VEGF-D are involved in lymphangiogenesis [14]. Little is known about the meaning of VEGF-B expression in tumours [11]. The family also include three tyrosine kinase receptors (TKR): VEGFR-1 (Flt-1), VEGFR-2 (KDR) and VEGFR-3 (Flt-4). The VEGFR-1 (vascular endothelial growth factor receptor 1) binds to VEGF-A and -B, VEGFR-2 binds to VEGF-A, -C and -D and VEGFR-3 binds to VEGF-C and -D [15]. The increased expression of VEGF family in tumours has been associated with poor prognosis or increased risk of recurrence or metastasis in several types of cancers [16] and, currently, several pro- or anti-angiogenic drugs have been approved by the FDA or are in clinical trials [17]. Very recently, in advanced cervical cancer, the use of the humanized anti-VEGF monoclonal antibody bevacizumab, in combination with chemotherapy, showed and improvement in patients’ overall survival [18]. Importantly, the Warburg effect may contribute to vascular modulation as tumour acidity facilitates angiogenesis [19] and lactate, also a contributor of tumour acidity, may itself induce VEGF [20].

Cervical cancer is still highly prevalent in developing and poor countries and studies on the biology related to the carcinogenesis associated with the high risk HPV infection is critical to understand the behaviour of these tumours and propose alternatives for their therapy and control [21].

Therefore, the present study was designed to evaluate the immunohistochemical expression of a variety of metabolic and vascular markers as common targets of the hypoxic microenvironment, assess the associations between them and associate their expression with the clinical and pathological behaviour of squamous cell carcinomas of the uterine cervix. Additionally, we also characterized the expression of the metabolic markers in *in vitro* 3D culture of keratinocytes transduced with HPV16 oncogenes or transfected with HPV16 or HPV18 full-length genome.

## Methods

### Human uterine cervix samples

The human biological samples analysed included 129 formalin-fixed paraffin embedded samples retrieved from the files of Pathology Division of Barretos Cancer Hospital, São Paulo, Brazil, which included 28 cases of chronic cervicitis, 34 cases of cervical intraepithelial neoplasia grade I (CIN 1, herein designated as low-grade squamous intraepithelial lesion - LSIL), 29 cases of cervical intraepithelial neoplasia grades II and III (CIN2/3, herein designated as high-grade squamous intraepithelial lesions - HSIL) and 38 cases of squamous cells carcinoma (SCC) of the uterine cervix. SCC samples were organized into one tissue microarray (TMA), with 96 tumour cores (one mm diameter), also including several control samples (kidney and placenta). Each case was represented in the TMA by at least two cores. Clinicopathological data of the patients included age at diagnosis, tumour size, histological grade, clinical stage, vascular invasion, lymph-node and distant metastasis, recurrence and survival; however, since histological grade, vascular invasion, lymph-node and distant metastasis, recurrence and survival were poorly represented in one of the groups, only age at diagnosis (mean 48 years), tumour size (mean 2 cm) and FIGO clinical stage (1 IA2 case, 32 IB1 cases, 4 IIA cases and 1 IIB case, grouped in clinical stage I and clinical stage II) were used for statistical analysis.

The ethics committee of Barretos Cancer Hospital approved this study. As retrospective study, the Ethical Committee does not require the written informed consent from the patients. The patients previously supplied consent for the samples to be used for research purposes.

### Epithelial raft cultures

Low passage-pooled neonatal foreskin keratinocytes (Lonza Walkersville, Inc., Walkersville, MD) were grown in keratinocyte serum-free medium (Invitrogen, Frederick, MD). Cells were acutely infected with recombinant pLXSN retrovirus vectors either empty or containing HPV16 E6, E7 or both E6/E7, and expressing the neomycin resistance marker. After 24 hours, the cells were selected with 250 μg/ml of G418 for two days, when 100% of mocked infected controls were dead. Recombinant pLXSN retrovirus vectors were kindly provided by Dr. Denise Galloway (Fred Hutchinson Cancer Research Center, Seattle, WA) and are described elsewhere [22]. The cell lines FK16A and FK18B were established by transfection of primary human foreskin keratinocytes (FK) with the entire HPV16 and HPV18 genome and cultured for different passage number as previously described [23]. All cells were used to seed epithelial rafts cultures, as previously described [23].

### Immunohistochemistry

MCT1 and CD147 immunohistochemistry was performed according to the avidin-biotin-peroxidase complex method (R.T.U. VECTASTAIN Elite ABC Kit (Universal), Vector Laboratories, Burlingame, CA), as previously described [24]. Immunohistochemistry for MCT2, MCT4, GLUT1 and CAIX was performed according to the streptavidin-biotin-peroxidase complex principle (Ultravision Detection System Anti-polyvalent, HRP, Lab Vision Corporation, Fremont, CA), as previously described [25, 26]. Negative controls were performed by the use of appropriate serum controls for the primary antibodies (N1698 and N1699, Dako, Carpinteria, CA). Colon carcinoma tissue was used as positive control for MCT1, and MCT4 and CD147, kidney was used for MCT2, head and neck cancer was used for GLUT1 and stomach was used for CAIX. Tissue sections were counterstained with hematoxylin and permanently mounted. Please refer to Table 1 for detailed aspects for each antibody used.

**Table 1:** **Detailed aspects for each antibody used in immunohistochemistry**

Protein	Antigen retrieval	Antibody	Antibody dilution and incubation time
**MCT1**	Citrate buffer (0.01 M, pH = 6), 98°C, 20’	AB3538P	1:200, overnight
		Chemicon International	
**MCT2**	Citrate buffer (0.01 M, pH = 6), 98°C, 20’	sc-50322	1:200, 2 hours
		Santa Cruz Biotechnology	
**MCT4**	Citrate buffer (0.01 M, pH = 6), 98°C, 20’	sc-50329	1:500, 2 hours
		Santa Cruz Biotechnology	
**CD147**	EDTA (1 mM, pH = 8), 98°C, 20’	sc-71038	1:400, overnight
		Santa Cruz Biotechnology	
**GLUT1**	Citrate buffer (0.01 M, pH = 6), 98°C, 20’	ab15309-500	1:500, 2 hours
		AbCam	
**CAIX**	Citrate buffer (0.01 M, pH = 6), 98°C, 20’	ab15086	1:2000, 2 hours
		AbCam	
**VEGF-A**	CC1 (pH = 8,2) Ventana	VG-1	1:200, 60 minutes
		AbCam	
**VEGF-C**	ptLink (pH = 9) Dako	18-2255	1:200, 60 minutes
		Invitrogen	
**VEGF-D**	ptLink (pH = 9) Dako	ab63068	1:50, 60 minutes
		AbCam	
**VEGFR-1**	ptLink (pH = 9) Dako	ab9540	1:300, 60 minutes
		AbCam	
**VEGFR-2**	ptLink (pH = 9) Dako	ab2349	1:100, 60 minutes
		AbCam	
**VEGFR-3**	ptLink (pH = 9) Dako	ab72240	1:50, 60 minutes
		AbCam	

For VEGFs immunohistochemical analyses, the immunohistochemical staining was performed automatically with Ventana Benchmark^®^ XT (Ventana Medical Systems, Tucson, AZ) following the manufacturer’s guidelines and then counterstained with hematoxylin and permanently mounted. Negative controls were obtained by omitting the primary antibody incubation step and human tonsils were used as positive control. Please refer to Table 1 for detailed aspects for each antibody used.

### Immunohistochemical evaluation

Sections were scored semi-quantitatively for plasma membrane expression (metabolic markers) and cytoplasmic expression (vascular markers) in cancer cells as follows: 0: 0% of immunoreactive cells; 1: <5% of immunoreactive cells; 2: 5-50% of immunoreactive cells; and 3: >50% of immunoreactive cells. Also, intensity of staining was scored semi-qualitatively as follows: 0: negative; 1: weak; 2: intermediate; and 3: strong. The final score was defined as the sum of both parameters (extent and intensity), and grouped as negative (score 0 and 2) and positive (score 3–6), as previously described [24]. Protein expression in other cellular localizations was also assessed. Two independent observers (AL-F and EAG) performed immunohistochemical evaluation blindly and discordant results were discussed in a double-head microscope in order to determine the final score.

### Statistical analysis

Data were stored and analysed using the IBM SPSS Statistics software (version 20, IBM Company, Armonk, NY). All comparisons were examined for statistical significance using Pearson’s chi-square (χ^2^) test and Fisher’s exact test (when n < 5). The threshold for significant *p* values was established as *p* < 0.05.

## Results

### Human samples

**Figure 1:**
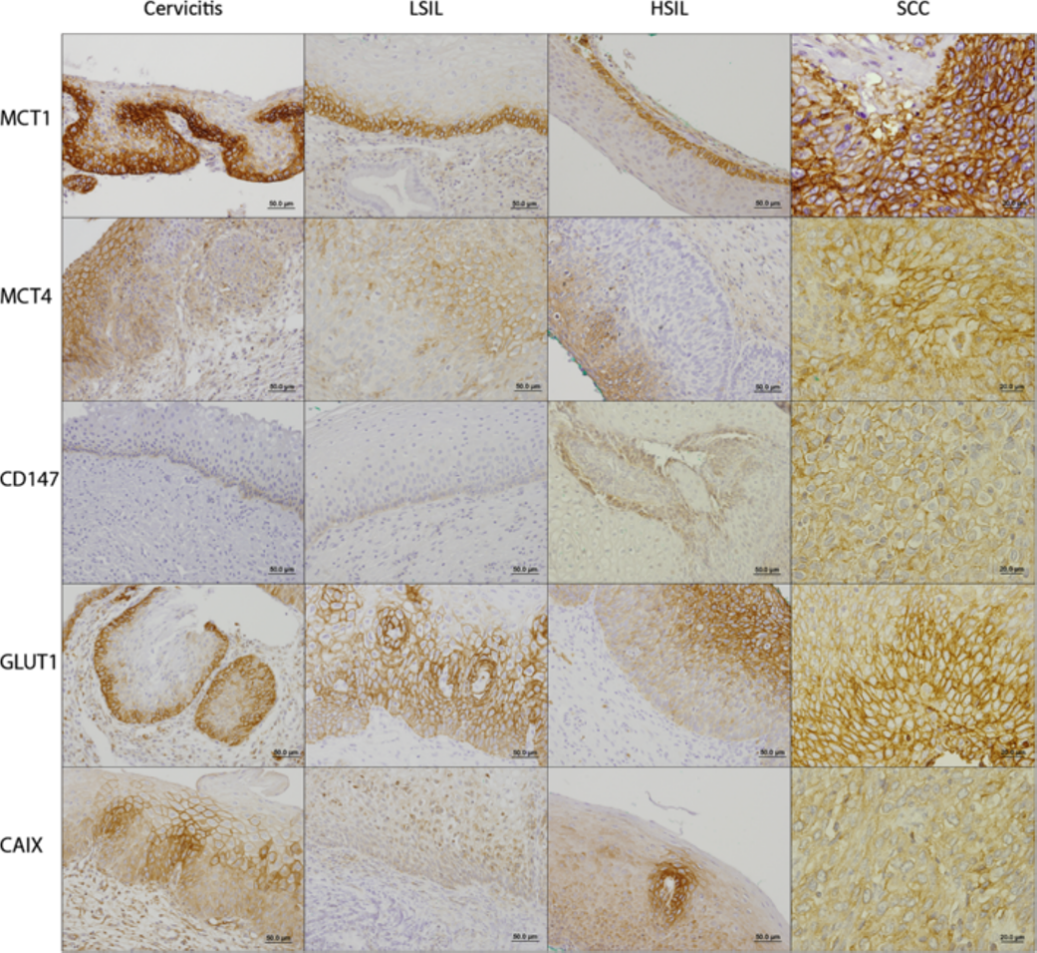
**Immunohistochemical expression of MCT1, MCT4, CD147, GLUT1 and CAIX in cervical samples.** All the proteins were more importantly found in the plasma membrane of cells.

The immunohistochemical evaluation showed that all the metabolic markers can be expressed in the cytoplasm, in the plasma membrane or exhibit both localizations. Figure 1 shows microphotographies representative of the expression of each protein, in each lesion. Importantly, only plasma membrane expression was considered for statistical analysis, as plasma membrane is required for the activity of the proteins studied as plasma membrane transporters or pH regulators.

Table 2 summarizes the expression frequencies at the plasma membrane of the metabolic markers MCT1, MCT4, CD147, GLUT1 and CAIX in cervicitis and from low-grade lesions towards SCC. MCT1 showed a gradual increase in its expression frequency along progression, however, without statistical significance. In opposition, the increase of MCT4 frequency was significantly higher in SCC, when compared with cervicitis, LSIL and HSIL (*p* = 0.005). CD147 followed a pattern of expression similar to MCT1, also without statistical significance, while GLUT1 was significantly increased in malignant lesions when compared with non-malignant lesions (*p* = 0.025). CAIX was significantly different among all the lesion groups (*p* = 0.004), but with no gradual increase/decrease along progression to malignancy.

**Table 2:** **Frequency of expression of the metabolic markers along progression to malignancy in cervical samples**

	***MCT1***			***MCT4***			***CD147***			***GLUT1***			***CAIX***		
	n	Positive (%)	***p***	n	Positive (%)	***P***	n	Positive (%)	***p***	n	Positive (%)	***p***	n	Positive (%)	***p***
			0.252			**0.005**			0.071			**0.025**			**0.004**
**Cervicitis**	**28**	6 (21.4)		**27**	6 (22.2)		**24**	0 (0.0)		**24**	12 (50.0)		**26**	11 (42.3)	
**LSIL**	**33**	9 (27.3)		**30**	8 (26.7)		**34**	2 (5.9)		**34**	29 (85.3)		**34**	4 (11.8)	
**HSIL**	**28**	9 (32.1)		**28**	6 (21.4)		**29**	2 (6.9)		**29**	19 (65.5)		**29**	8 (27.6)	
**SCC**	**34**	15 (44.1)		**33**	19 (57.6)		**32**	6 (18.8)		**37**	28 (75.7)		**33**	17 (51.5)	

Note: the number of lesion samples analysed for each marker varies due to loss of material during IHC procedure or lack of representativity of the lesion in the sample.

**Figure 2:**
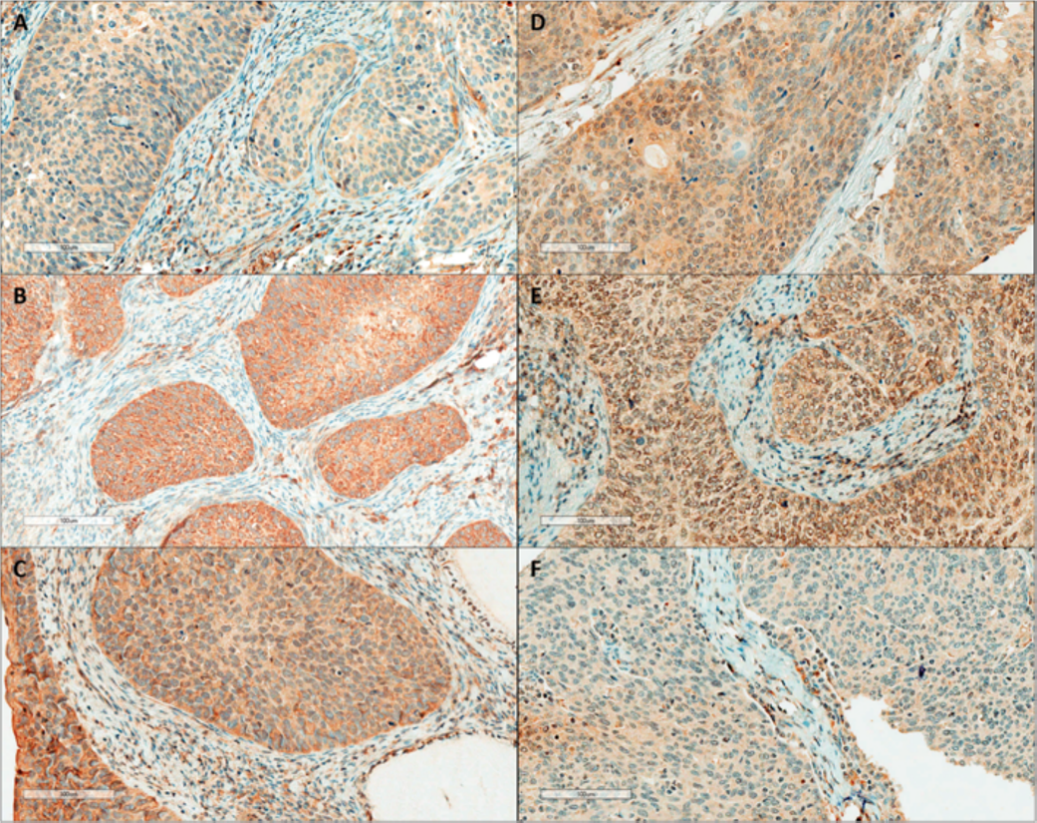
**Immunohistochemical expression of VEGF family in SCC samples. (A)** VEGF-A; **(B)** VEGF-C; **(C)** VEGF-D; **(D)** VEGFR-1; **(E)** VEGFR-2; and **(F)** VEGFR-3.

The co-expression between the different markers was also analysed. Considering all the human uterine cervix samples, MCT4 was significantly co-expressed with both CD147 and CAIX (*p* = 0.030 and *p* = 0.014, respectively), but not with GLUT1, while MCT1 was not significantly co-expressed with none of these three proteins (data not shown). Concerning the VEGF family members, overall, these proteins, with the exception of VEGFR-1 and VEGFR-2, presented a high intensity and extension of expression (Figure 2). No significant co-expression was observed between the expression of the ligands and the receptors (data not shown). When evaluating the possible co-expression between the metabolic and the vascular markers, a significant association between absence of MCT1 and presence of VEGF-A was found (Table 3, *p* = 0.013). To note, VEGF-D was not included in the latter analysis, as only 1 case was negative.

**Table 3:** **Association between the metabolic markers and the vascular markers in SCC samples**

	***VEGF-A***			***VEGF-C***			***VEGFR-1***			***VEGFR-2***			***VEGFR-3***		
	n	Positive (%)	***p***	n	Positive (%)	***p***	n	Positive (%)	***p***	n	Positive (%)	***p***	n	Positive (%)	***p***
**MCT1**			**0.013**			1.000			0.370			0.426			0.722
Negative	**17**	16 (94.1)		**19**	18 (94.7)		**19**	2 (10.5)		**19**	14 (73.7)		**18**	6 (33.3)	
Positive	**15**	8 (53.3)		**15**	14 (93.3)		**15**	4 (26.7)		**15**	13 (86.7)		**15**	4 (26.7)	
**MCT4**			0.108			0.172			0.209			0.670			1.000
Negative	**12**	11 (91.7)		**14**	12 (85.7)		**14**	1 (7.1)		**14**	12 (85.7)		**14**	4 (28.6)	
Positive	**19**	12 (63.2)		**19**	19 (100.0)		**19**	5 (26.3)		**19**	14 (73.7)		**19**	6 (31.6)	
**CD147**			0.645			1.000			1.000			1.000			0.314
Negative	**24**	18 (75.0)		**26**	25 (96.2)		**26**	5 (19.2)		**26**	20 (76.9)		**26**	6 (23.1)	
Positive	**6**	4 (66.7)		**6**	6 (100.0)		**6**	1 (16.7)		**6**	5 (83.3)		**6**	3 (50.0)	
**GLUT1**			0.390			0.553			0.340			1.000			0.205
Negative	**8**	7 (87.5)		**9**	9 (100.0)		**9**	3 (33.3)		**9**	7 (77.8)		**8**	4 (50.0)	
Positive	**27**	18 (66.7)		**26**	23 (88.5)		**26**	4 (15.4)		**26**	21 (80.8)		**25**	6 (24.0)	
**CAIX**			0.685			0.485			0.175			0.398			0.909
Negative	**15**	12 (80.0)		**16**	16 (100.0)		**16**	1 (6.2)		**16**	14 (87.5)		**16**	5 (31.2)	
Positive	**16**	11 (68.8)		**17**	15 (88.2)		**17**	5 (29.4)		**17**	12 (70.6)		**17**	5 (29.4)	

Results from the analysis of the expression of the different proteins and the available clinicopathological data of SCC is shown in Table 4. The only associations found were between both MCT4 and CD147 and patients younger at diagnosis (*p* = 0.029 and *p* = 0.024, respectively). Additional statistical analysis was performed (data not shown), assembling groups with different patterns of expression, by combining positivity for more than one protein (for example, co-expression of MCT1 with CD147 and VEGF-A). Importantly, co-expression of MCT1 with GLUT1 and CAIX was significantly associated with clinical stage, as stage I presented three (10.7%) positive cases and stage II three (60.0%) positive cases (*p* = 0.031).

**Table 4:** **Association of the metabolic and vascular markers expression with the clinicopathological variables in SCC samples**

	***MCT1***			***MCT4***			***CD147***			***GLUT1***			***CAIX***		
	n	Positive (%)	***p***	n	Positive (%)	***p***	n	Positive (%)	***p***	n	Positive (%)	***p***	n	Positive (%)	***p***
Age*			0.901			**0.029**			**0.024**			0.711			0.208
> 48	**14**	6 (42.9)		**14**	5 (35.7)		**14**	0 (0.0)		**15**	12 (80.0)		**14**	9 (64.3)	
≤ 48	**20**	9 (45.0)		**19**	14 (73.7)		**18**	6 (33.3)		**22**	16 (72.7)		**19**	8 (42.1)	
Tumour size*			0.644			0.600			1.000			0.624			1.000
≤ 2 cm	**11**	4 (36.4)		**11**	7 (63.6)		**11**	2 (18.2)		**14**	10 (71.4)		**11**	4 (36.4)	
> 2 cm	**6**	3 (50.0)		**6**	5 (83.3)		**6**	1 (16.7)		**7**	6 (85.7)		**6**	2 (33.3)	
**Clinical stage**			0.634			0.138			1.000			0.307			0.335
I	**29**	12 (41.4)		**28**	18 (64.3)		**27**	5 (18.5)		**32**	23 (71.9)		**28**	13 (46.4)	
II	**5**	3 (60.0)		**5**	1 (20.0)		**5**	1 (20.0)		**5**	5 (100.0)		**5**	4 (80.0)	
	***VEGF-A***			***VEGF-C***			***VEGFR-1***			***VEGFR-2***			***VEGFR-3***		
	**n**	**Positive (%)**	***p***	**n**	**Positive (%)**	***p***	**n**	**Positive (%)**	***p***	**n**	**Positive (%)**	***p***	**n**	**Positive (%)**	***p***
Age*			0.270			1.000			0.681			1.000			0.455
> 48	**13**	11 (84.6)		**14**	13 (92.9)		**14**	2 (14.3)		**14**	11 (78.6)		**14**	3 (21.4)	
≤ 48	**23**	15 (64.2)		**22**	20 (90.9)		**22**	5 (22.7)		**22**	18 (81.8)		**19**	7 (36.8)	
Tumour size*			0.624			1.000			0.517			0.557			0.600
≤ 2 cm	**14**	10 (71.4)		**13**	12 (92.3)		**13**	3 (23.1)		**13**	11 (84.6)		**11**	4 (36.4)	
> 2 cm	**7**	6 (85.7)		**6**	6 (100.0)		**6**	0 (0.0)		**6**	4 (66.7)		**6**	1 (16.7)	
**Clinical stage**			0.603			0.370			0.559			0.244			0.627
I	**31**	23 (74.2)		**31**	29 (93.5)		**31**	7 (22.6)		**31**	26 (83.9)		**28**	8 (28.6)	
II	**5**	3 (60.0)		**5**	4 (80.0)		**5**	0 (0.0)		**5**	3 (60.0)		**5**	2 (40.0)	

*Mean values were used for age and tumour size cut-off.

### Epithelial organotypic rafts cultures

**Figure 3:**
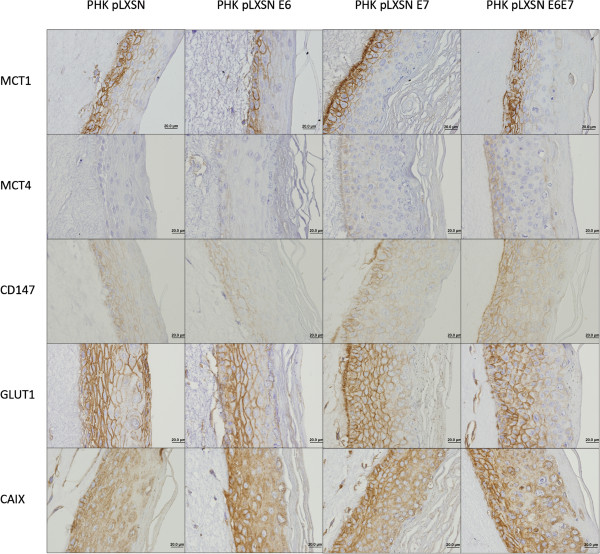
**Expression of metabolic proteins after transduction with HPV16 oncogenes.** The expression of MCT1, MCT4, CD147, GLUT1 and CAIX was evaluated by immunohistochemistry in *in vitro* 3D cultures of keratinocytes transduced with HPV16 oncogenes.

**Figure 4:**
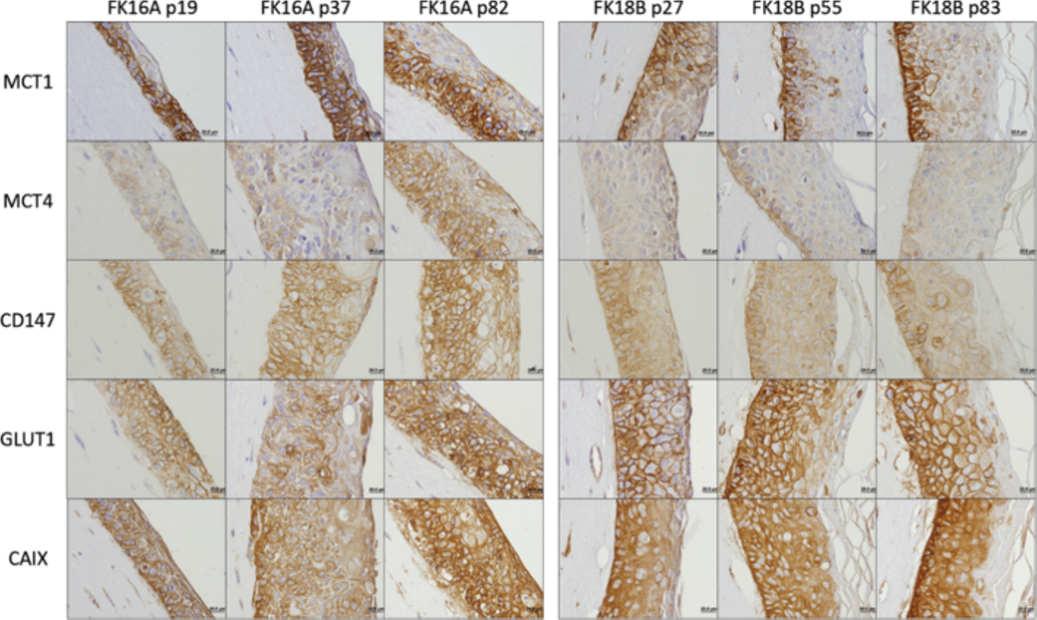
**Expression of metabolic proteins after transfection with HPV16 or HPV18 full genome.** The expression of MCT1, MCT4, CD147, GLUT1 and CAIX was evaluated by immunohistochemistry in *in vitro* 3D cultures of keratinocytes transfected with HPV16 or HPV18 full genome.

Immunohistochemical expression analysis of the different key metabolic proteins was also performed using epithelial organotypic rafts cultures obtained from low passage keratinocytes expressing HPV16 E6 and/or E7 expression, as well as from PHK transfected with HPV16 or 18 full-length genomes and grown during different passages before raft culture seeding (Figures 3 and 4). Expression of HPV oncoproteins did not influence MCT1 expression; however, E7 expression upregulates MCT4 in the basal layer of the epithelium and increases the expression of CD147 throughout the epithelium thickness. Importantly, raft cultures from high passage HPV16-expressing cells, which resemble severe dysplasia in vivo, present an increase in MCT4 expression and the same was observed for CD147 in HPV16 and 18-transfected cells, with no apparent change in MCT1 expression. Concerning GLUT1, neither E6 nor E7 seem to affect its expression, while high passage after HPV16 transfection seems to stimulate GLUT1 expression. Finally, CAIX seems to be increased after E6 and/or E7 expression, while passage number in HPV16-transfected cells also increased the expression intensity of this protein.

## Discussion

Recently, the cancer metabolic adaptations have been included in the well-known Hallmarks of Cancer [27]. Aerobic glycolysis, although being a much less efficient energetic pathway than mitochondrial oxidative phosphorylation, is a successful adaptation to hypoxic microenvironment, allows cancer cells to use the most abundant extracellular nutrient, glucose, to produce ATP, and excess pyruvate provides a source for anabolic substrates essential for biosynthetic pathways [28]. Hypoxia conditions, which are known to induce the glycolytic phenotype, are associated with altered expression of MCTs [29, 30]. Some evidence points at hypoxia-mediated increase of MCT4, but not MCT1 [29], while other points at regulation of both MCT isoforms by hypoxia [30]. This controversial data on MCT regulation by hypoxia, especially MCT1, instigated us to further investigate this association between MCTs and hypoxia, by analysing the co-expression of these proteins with GLUT1 and CAIX, which are targets of HIF-1α [8, 9]. It is important to mention, however, that, as described in few studies, both HIF-1α and GLUT1 expression may not be quantitatively correlated to hypoxia in cervical cancer [31, 32].

Firstly, the pattern of expression of these markers was analysed concerning the progression from cervicitis, through LSIL, HSIL and finally SCC. In accordance to the previous publication from our group using a different cervical cancer series [33, 34], both MCT1 and MCT4 showed an increased expression towards malignancy, although only significant for MCT4. Importantly, in this study, the tendency for an increase in plasma membrane expression of MCT1 and CD147 was more evident than in the previous studies. Unfortunately, due to the low number of cases these increases were not significant but the predominant membranous pattern of concomitant immunoreaction of both markers suggest a potential more effective metabolic activity, which can play a role in cancer development. GLUT1 expression, although with significantly different frequencies of expression among the lesions, did not follow a gradual increase towards malignancy. Previous results from others have described a progressive increase of GLUT1 from normal cervical tissue, to precursor lesions and SCC [35, 36]. CAIX showed a gradual expression frequency in cervical lesions, according to lesion grade. Notably, its expression in cervicitis was almost as frequent as in SCC. This observation may be explained by the association between hypoxia and inflammation, as pro-inflammatory cytokines like TNF-α can stimulate HIF-1α activity [37] and, as a consequence, induce CAIX expression. Therefore, ideally, normal cervical samples instead of cervicitis samples should be used, however, these samples are difficult to obtain as biopsies are only performed when there is suspicion of lesion.

When comparing the expression of MCT1 with its chaperone CD147 and the hypoxia-induced GLUT1 and CAIX, no significant associations were found. However, for CD147, this lack of association may be associated with the low number of cases. Additionally, as hypothesised in other studies [33, 38–40], other chaperones not yet identified should be involved in MCT trafficking to the plasma membrane. In opposition, we found a significant co-expression between MCT4 and both CD147 and CAIX, and a tendency for co-expression with GLUT1. These results go along with the hypothesis that MCT4, but not MCT1, is associated with hypoxia [29] and also with the hyper-glycolytic (GLUT1) and acid-resistant phenotype (CAIX) of cancer cells. Therefore, MCT4 would be important for the efflux of lactate and protons in hyper-glycolytic cancer cells, allowing maintenance of the glycolytic flow and helping in the prevention of apoptosis resultant from low intracellular pH.

Overall, the results obtained with the human samples were confirmed *in vitro*, with the 3D culture of keratinocytes expressing HPV genes. At the best of our knowledge, this is the first study that attempted to evaluate metabolic markers in epithelial rafts expressing E6 and E7 HPV oncogenes. In the literature, studies by others show contradictory results about the possible effect of HPV infection in the metabolic behaviour of cancer cells. In one hand, HPV infection has been demonstrated to stabilize HIF-1α expression [41, 42]. As HIF-1α is the major regulator of the Warburg effect [7], one could conclude that HPV infection will stimulate glycolysis enhancing the expression of glycolytic-associated proteins. However, in the other hand, it was described that HPV16 E7 oncoprotein expression stimulates glutaminolysis, decreasing glucose consumption and lactate production [43]. In the present study, we found that both MCT4 and CD147 were stimulated in E7 expressing cells, while CAIX was stimulated by E6 and E7, alone or in combination, which is in accordance with the stimulation of the Warburg effect, as a result of HPV-dependent HIF-1α stabilization [41, 42]. However, both MCT1 and GLUT1 were not affected by E6 and/or E7 expression, which, for MCT1, once again, suggest that this protein may not be induced by HIF-1α, at least in this model. Besides, for GLUT1, this observation is in accordance with the results from the human samples as well as the previous results showing the stimulation of glutaminolysis, but not glycolysis, after HPV16 E7 oncoprotein expression [43]. This stimulation of glutaminolysis would also explain MCT4 increase, as glutaminolysis also leads to lactate production. Additionally, high passage HPV16 whole genome transfected cells present higher expression of MCT4, CD147, GLUT1 and CAIX, while HPV18 whole genome transfected cells present higher expression of CD147. As organotypic cultures obtained from HPV-positive cells at low passage number (FK16A p19 and FK18B p27) exhibit morphological alterations reminiscent of mild/moderate dysplasia while those obtained from the same cells at high passage number (p > 80) exhibit more severe dysplastic features, we can hypothesise that MCT4, CD147, GLUT1 and CAIX are increased during HPV-associated lesion.

It is well-known that tumour acidity facilitates cancer cell invasion behaviour [44]. Additionally, cancer acidosis is also associated with mutagenesis/clastogenesis, radioresistance and resistance to anthracyclines [2]. Although sharing its role as tumour acidifier with other sources of acidity like carbon dioxide, lactate has been proven to cause acidification of tumour microenvironment [45]. Importantly, lactate has other properties which contribute modestly to the malignant behaviour of cancer cells; exogenous lactate was demonstrated to increase cellular motility [46], induce VEGF [20], as well as hyaluronan and its receptor CD44, which are molecules involved in the process of cancer invasion and metastisation [47]. As a result, lactate can enhance the malignant phenotype of tumour cells, contributing to the association of high tumour lactate concentrations with incidence of metastases, tumour recurrence, patient survival [48] and radioresistance [49]. Following this rationale, we analysed the expression of vascular factors in the SCC samples and verify its co-expression with the metabolic markers. Firstly, we observed an overall high expression of the members of the VEGF family, especially the ligands. This is in accordance with previous publications from other authors, where high expression of VEGF family members is described in cervical cancer [50]. As a result of this high expression, as well as the low number of cases, no significant associations were found when analysing the possible co-expression of VEGF ligands with the receptors. Interestingly, when analysing the co-expression of the metabolic markers with the vascular markers, we found an inverse association between MCT1 and VEGF-A. As described for colorectal cancer with MCT4 and VEGF-A [51], it is possible that these proteins are involved in different stages of tumour progression, where VEGF-A is involved in the early stages of tumour growth while MCTs, in this case MCT1, appears as the tumour enlarges and may contribute to further tumour infiltration and growth. Our data on association with tumour size do not support this hypothesis, however, the number of cases with information on tumour size is even lower, with only 21 cases available for analysis, losing statistical power. Further studies, with a higher number of samples, are required to validate this inverse association and additional experiments are needed to elucidate the molecular mechanisms, if any, behind this event.

The clinicopathological significance of the metabolic markers analysed in the present study has been previously evaluated by us and others [33–36, 52–55]. Although we were not able to found any associations with the clinicopathological data of SCC in our previous studies with MCTs and CD147 [33, 34], other studies have found association of CD147, GLUT1 and CAIX expression with lymph-node metastasis [35, 52, 54], while CAIX was also associated with advanced tumour stage, greater invasion depth, undifferentiated tumour grade [52], as well as distant metastasis in early-stage cervical cancer [53]. Also, absence of GLUT1 was shown to significantly increase the likelihood of metastasis-free survival in locally advanced cervical carcinoma but not of disease-free or recurrence-free survival [55]. However, another study showed that, despite the increased expression of HIF-1α, GLUT1 and CAIX in cervical cancer, only HIF-1α was associated with poor prognostic variables like lymph node metastasis and overall survival [36]. In the present study, we found significant association of both MCT4 and CD147 with younger patients at time of diagnosis. Cervical cancer is a cancer of late onset, as tumours take years to develop since high-risk HPV type infection, which is a necessary cause for the development of these tumours [56]. Therefore, we hypothesize that MCT4 and CD147 expression may provide to the abnormal cells a survival advantage and a potential for progression that allow tumours to arise earlier. Additionally, we also found significant association between co-expression of MCT1/GLUT1/CAIX and higher clinical stage, which is in accordance with the more aggressive behaviour of hyper-glycolytic and acid-resistant cancer cells. In respect to VEGF family members, although some studies show the clinicopathological value of VEGF family members in cervical cancer [50, 57, 58], in the present study, we found no association of VEGFR family members and the clinicopathological variables available, which, at least for VEGF-A, is in accordance with another previous study [59]. Further studies, with larger and best characterized squamous cervical cancer series, are required to rigorously confirm these results, as well as evaluate possible associations with survival.

## Conclusions

In the present study, we confirmed the induction of MCT4 in the progression towards malignancy in cervical squamous cells carcinoma, pointing at this protein as a promising therapeutic target. This increase seems to be associated with hypoxia or HPV-induced HIF-1α stabilization and is accompanied by CD147 and CAIX, but not GLUT1, expression. With this evidence, we take a small step forward regarding MCT behaviour in response to hypoxic conditions/HIF-1α, providing additional evidence for the regulation of MCT4, but not MCT1, under these conditions. Also, we show an inverse association between MCT1 and the major angiogenic factor VEGF-A, which should be further explored in a larger cervical cancer series, including also other histological types like adenocarcinomas, as well as in series from other tumours types.

## Abbreviations

CAIX: Carbonic anhydrase IX
GLUT1: Glucose transporter 1
HIF-1α: Hypoxia inducible factor 1 alpha
HSIL: High-grade squamous intraepithelial lesions
LSIL: Low-grade squamous intraepithelial lesion
MCTs: Monocarboxylate transporter
SCC: Squamous cells carcinoma
TMA: Tissue microarray
VEGF: Vascular endothelial growth factor
VEGFR: Vascular endothelial growth factor receptor.
